# LC-MS-based Metabolomics of Xenobiotic-induced Toxicities

**DOI:** 10.5936/csbj.201301008

**Published:** 2013-03-12

**Authors:** Chi Chen, Sangyub Kim

**Affiliations:** a Department of Food Science and Nutrition, University of Minnesota, St. Paul, MN 55108, United States

**Keywords:** metabolomics, LC-MS, xenobiotic, xenobiotic-induced toxicity, biomarker

## Abstract

Xenobiotic exposure, especially high-dose or repeated exposure of xenobiotics, can elicit detrimental effects on biological systems through diverse mechanisms. Changes in metabolic systems, including formation of reactive metabolites and disruption of endogenous metabolism, are not only the common consequences of toxic xenobiotic exposure, but in many cases are the major causes behind development of xenobiotic-induced toxicities (XIT). Therefore, examining the metabolic events associated with XIT generates mechanistic insights into the initiation and progression of XIT, and provides guidance for prevention and treatment. Traditional bioanalytical platforms that target only a few suspected metabolites are capable of validating the expected outcomes of xenobiotic exposure. However, these approaches lack the capacity to define global changes and to identify unexpected events in the metabolic system. Recent developments in high-throughput metabolomics have dramatically expanded the scope and potential of metabolite analysis. Among all analytical techniques adopted for metabolomics, liquid chromatography-mass spectrometry (LC-MS) has been most widely used for metabolomic investigations of XIT due to its versatility and sensitivity in metabolite analysis. In this review, technical platform of LC-MS-based metabolomics, including experimental model, sample preparation, instrumentation, and data analysis, are discussed. Applications of LC-MS-based metabolomics in exploratory and hypothesis-driven investigations of XIT are illustrated by case studies of xenobiotic metabolism and endogenous metabolism associated with xenobiotic exposure.

## Introduction

Exposure to exogenous chemicals (xenobiotics) via diet, environment, or medication is inevitable in all living creatures and interactions between xenobiotics and a biological system are bidirectional. On one hand, xenobiotics are actively processed by a biological system through absorption, distribution, metabolism, and excretion. On the other hand, a biological system can be significantly affected by xenobiotics, especially when subjected to a high-dose or repeated exposure. Among these interactions, metabolic activities, which include both xenobiotic metabolism and endogenous metabolism, play central roles in determining the biological consequences of xenobiotic exposure, especially in xenobiotic-induced toxicities (XIT). Numerous toxicological studies have demonstrated that the formation of reactive metabolites and the disruption of endogenous metabolism are major contributors to the initiation and progression of XIT [1, 2]. Therefore, examining metabolic events elicited by xenobiotic exposure is indispensable for studying the mechanisms of XIT.

Because XIT-associated metabolic changes occur at gene, protein (enzyme), and metabolite levels, biochemical and chemical analyses of genes, proteins, and metabolites are commonly conducted to acquire mechanistic information on the metabolic activities in XIT.

Compared to the changes in genes and proteins, which mainly indicate the potential of physiological changes, changes in metabolites reflect the real metabolic consequences of XIT due to the fact that metabolites are the end products of metabolic activities. Traditional metabolite analysis in toxicological studies is commonly driven by hypothesis and usually has specific targets. This approach has its merit in unraveling mechanisms in many toxic events, but has its limitation, especially in identifying novel and unexpected metabolic activities [3, 4]. In XIT, biotransformation of xenobiotic and disruption of endogenous metabolism, in general, are far more complex than a few metabolites and pathways examined by targeted metabolite analysis. Thus, a more comprehensive and non-targeted approach is needed to obtain the global view of XIT-elicited metabolic events. As a result of this need, metabolomics has gradually become a preferred analytical approach to exam the metabolic activities in XIT [5, 6].

Based on high-throughput data acquisition and robust data analysis, metabolomics is capable of monitoring hundreds of metabolites simultaneously in a given biological sample and detecting subtle changes in a large dataset. Technical advances in bioanalysis, chemometrics, and bioinformatics have made metabolomics an important component of systems biology, complementing genomics, transcriptomics and proteomics [7–9]. One of the original incentives behind the development of metabolomics techniques was to exam the metabolic events in XIT [10, 11]. However, owing to its clear advantage over traditional targeted metabolite analysis in complex biological matrices, applications of metabolomics have been expanded to all aspects of biological science, including plant and animal biology [12], microbiology [13], and disease diagnosis [14].

A variety of detection methods have been adopted for metabolite analysis in metabolomics, including electrochemical array [15, 16], infrared spectroscopy [17], nuclear magnetic resonance (NMR), and mass spectrometry (MS). Among these platforms, MS and NMR are the most widely used. The pros and cons of using NMR and MS techniques in metabolomics research have been discussed previously [18–20]. Compared to MS, NMR has clear advantages in two aspects: 1) its non-destructive nature reduces the need for sample preparation; 2) its indiscriminant nature in detection offers broad coverage and high-throughput capacity in analyzing diverse chemical species. However, NMR-based metabolomics is limited by the lack of sensitivity in detecting large numbers of low-abundance metabolites in biological matrices and this leads to repetitive identification of several “usual suspect” metabolites as the key biomarkers in many NMR-based metabolomics studies [21]. Nevertheless, the advent of NMR instruments with stronger magnetic fields should increase the sensitivity of NMR metabolomics [22].

Owing to the rapid progresses in MS instrumentation during the past decade, MS has achieved much greater sensitivity than NMR in detecting small-molecule metabolites and thus can provide more comprehensive information on metabolite profile [23]. However, compared to NMR, MS also has several drawbacks, such as the need for sample preparation, irrecoverable sample loss during MS analysis, biased metabolite detection caused by inconsistent ionization efficiency in the MS instruments, and the lack of automatic metabolite identification [24]. Therefore, selection of MS or NMR for metabolomic investigation is commonly determined by consideration of the pros and cons of the technical platforms as well as availability of the instruments.

The method used to introduce prepared samples into the MS system significantly affects the results of MS detection. The shot-gun approach that directly infuses samples into the MS system has been successfully adopted for analyzing metabolites in tissue and lipid extracts [25]. Advantages of this approach include the efficiency in data acquisition and the avoidance of sample dilution during chromatographic separation. However, disadvantages are also apparent and include ion suppression in the ionization process and difficulty in distinguishing ions with the same molecular mass. Therefore, more commonly, the metabolites in samples are separated by chromatographic systems prior to MS analysis [26]. According to the chemical properties of samples, respective separation methods, including capillary electrophoresis, gas chromatography (GC), or liquid chromatography (LC) can be selected for metabolomic analysis. Capillary electrophoresis is highly efficient in separating polar and charged compounds based on their different migrating velocities in the electric field, but its low capacity in sample loading and poor sensitivity on non-polar compounds limit its application in comprehensive metabolite profiling [27]. GC and LC platforms are more commonly used for separating metabolites in biological samples. Compared to LC, GC has an advantage in resolution and reproducibility of chromatographic separations, which can facilitate metabolite identification and chemometric analysis. However, due to the incompatibility of GC columns with water and other polar solvents, multi-step sample preparation processes including solvent extraction, drying, and derivatization, are required to make samples volatile and this can significantly affect integrity of the sample metabolome [28]. In contrast, LC techniques have much better compatibility with water-based biofluids and tissue/cell extracts. In fact, the reduced need for sample preparation and the greater compatibility of LC with diverse metabolites have promoted its widespread adoption as the preferred separation tool in metabolomics. Hence, this review will mainly discuss the technical platform of LC-MS-based metabolomics and its applications in studying XIT.

## Technical platform of LC-MS based metabolomics

**Figure 1:**
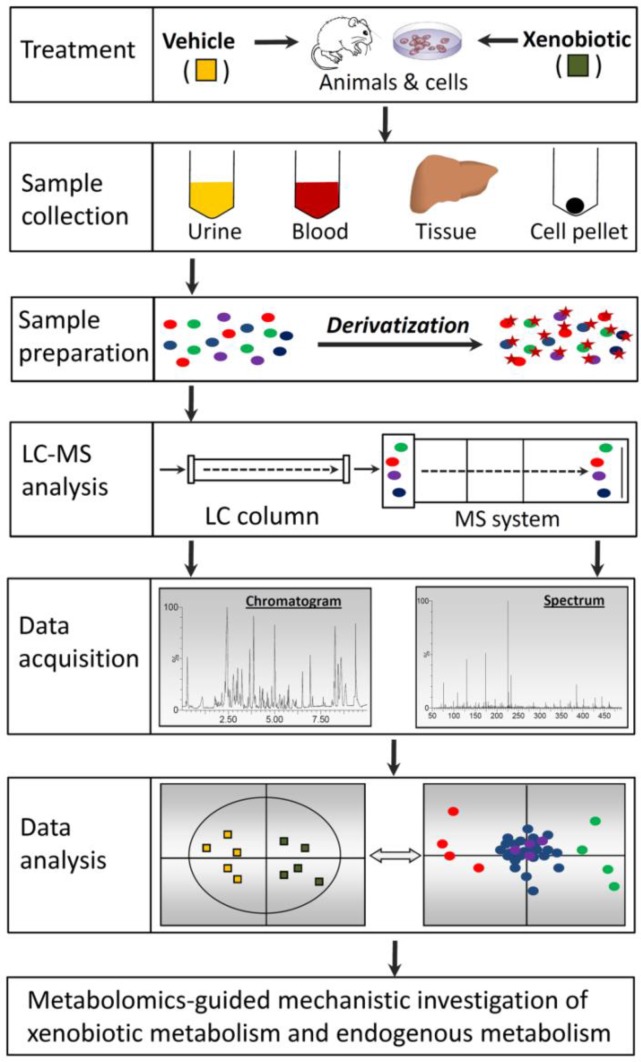
The work flow of untargeted LC-MS-based metabolomics. Samples from diverse sources need to be processed appropriately to make them compatible with LC-MS analysis. Chemical derivatization can be performed to facilitate the chromatographic separation of metabolites in the LC and increase the sensitivity of metabolite detection in the MS system. Chromatographic and spectral data are acquired by high-resolution LC-MS. Subsequent data processing, such as centroiding, deisotoping, filtering, and peak recognition, yields a data matrix constructed by sample identity, ion identity (RT and *m/z*), and ion abundance. Through data transformation and multivariate data analysis, a multivariate model can be established in which the scores plot illustrates the principal or latent components of the model as well as sample classification while the loadings plot presents the contribution of each ion to sample classification in the model.

The capacity of LC-MS-based metabolomics for identifying biomarkers and revealing mechanisms originates from its sophisticated technical platform. Recent advances in LC-MS-based metabolomics have been driven by the availability of diverse experimental models and the development of improved techniques for sample preparation, LC-MS analysis, data acquisition, and data analysis (Fig. 1).

### Experimental model

Selecting an appropriate experimental model is essential for acquiring the samples that can best reflect the metabolic changes in XIT. *In vitro* models, such as the incubations of xenobiotics with cell culture, tissue homogenates, or purified enzymes, can be adopted to examine hypothesis on xenobiotic-elicited metabolic events associated by specific tissue, cells, or enzymes. However, to reveal xenobiotic-induced global metabolic changes in a biological system, animals and humans are still the best sources for sample collection. Compared to animals, using humans as experimental subjects has clear advantage in clinical significance, but has disadvantages in other aspects: 1) in general, pre-treatment metabolic differences among human subjects are much greater than differences among experimental animals due to both internal and external factors, such as genetic polymorphism, age, and health status; 2) environment, diet, and life style vary greatly in human subjects, but are under strict control in experimental animals; 3) for XIT, toxic doses can be achieved in animal models, but not in humans under experimental conditions; 4) tissue samples are accessible in animal models, but rarely available in humans. Therefore, in many cases, animals are more robust experimental models for defining the mechanisms of XIT. Nevertheless, if under sound experimental design and sufficient number of experimental subjects, metabolomic investigation of xenobiotic exposure in humans still provides great opportunities for identifying metabolic biomarkers of XIT and verifying the observations in animal studies.

### Sample preparation

**Figure 2:**
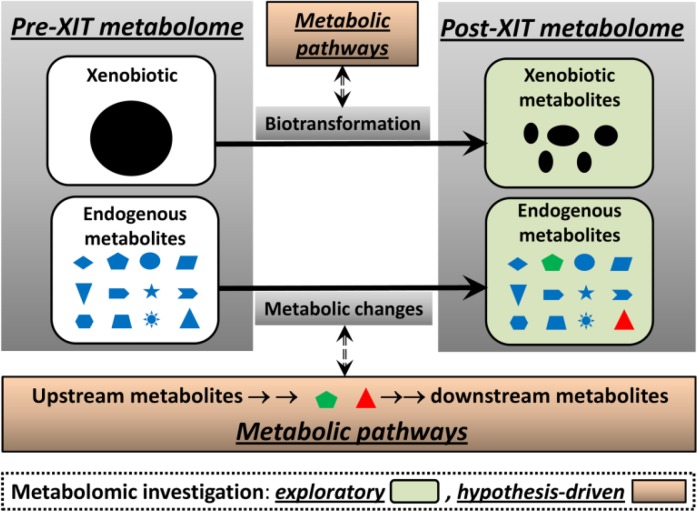
Metabolic events in XIT and potential targets of LC-MS-based metabolomics. Biotransformation of xenobiotics and xenobiotic-induced metabolic changes occur simultaneously during the initiation and progression of XIT. LC-MS-based metabolomics is not only able to identify the metabolites generated or affected by these events (exploratory investigation), but also capable of revealing the biochemical mechanisms underlying these events when combined with other experimental models and biochemical analyses (hypothesis-driven investigation).

To maximize the information available for LC-MS analysis, sample preparation has to be designed and performed based on the chemical and biochemical properties of the particular biological matrix of the samples (Fig. 2). Dramatic metabolic changes are usually associated with XIT and it is not uncommon for many XIT-associated metabolites to be vulnerable to further metabolic changes until the potential for change is terminated. Potential post-collection alterations include enzyme-mediated biochemical reactions and degradation due to unfavorable environmental conditions (high temperatures) or bacterial contamination. To reduce or avoid these changes prior to metabolomic analysis, rapid freezing in liquid nitrogen or quenching treatments are widely used for tissue and cell culture samples [29], while bactericides and cold traps are frequently used for urine collections.

Without appropriate processing, biological samples, including urine, blood, tissue, and cell culture, are not suitable for LC-MS analysis due to the biomatrices of these samples [30]. Sample processing aims to preserve integrity of the sample metabolome and also facilitate the detection of metabolites [31]. Basic sample processing procedures, such as removing proteins and particles, are required for all samples, while specific procedures are adopted according to the physical and chemical properties of samples as well as the aims of metabolomic analysis. For urine samples, the interference of its inorganic salt content with LC-MS analysis can be abated by dilution or solid phase extraction. Serum contains both water-soluble metabolites and large quantities of lipid species. Therefore, besides stringent protein removal, phase separation methods, such as Bligh's [32] for hydrophilic and lipophilic faction in serum, should be considered before LC-MS analysis. Subcellular fractionation of tissue and cell culture samples prior to phase separation can be conducted to identify distribution and changes of metabolites within specific intracellular organelles or compartments such as mitochondria and the cytosol. In multiple-step sample processing, adding internal standard is recommended to account for the experimental variances across samples.

Making samples more compatible for LC-MS analysis is not the only aim of sample preparation. Enhancing the sensitivity of metabolite detection is the other important aim of sample preparation. Metabolite concentration in samples and ionization efficiency in MS are two major factors determining the sensitivity of metabolite detection in LC-MS analysis. Low-abundance metabolites in samples can be concentrated by condensation, solvent extraction, or column extraction. However, for many metabolites, the barriers for their detection in LC-MS analysis are not their concentrations in samples, but their non-optimal performance in LC and MS systems, such as poor retention in LC column and insufficient ionization in MS [32]. To enhance the chromatographic and spectroscopic performance of these metabolites, one effective approach is to conduct chemical derivatization (Fig. 1). Chemical derivatization has been widely used in GC-MS analysis to improve separation, detectability and sensitivity of metabolite detection. The application of chemical derivatization in LC-MS analysis has greatly expanded in the recent years [33]. In general, derivatization reactions are designed based on the functional groups, such as amino, carboxyl, carbonyl, and hydroxyl moieties in the metabolites. Increased hydrophobicity and chargeability are two desired effects of chemical derivatization [34]. For example, amino acids are commonly derivatized by dansyl chloride [35]. The detection of organic acids is significantly enhanced through esterification of carboxyl group with amines, hydrazines, or alcohol, while detection of aldehydes and ketones is assisted by the formation of Schiff bases after derivatization reactions [36, 37].

### LC-MS analysis and data acquisition

The mobile phase and column of the LC system and the ion source and mass detector of the MS system (Fig. 1) are major components of LC-MS analysis that can significantly affect data quality. The mobile phase plays a critical role in metabolite separation in the LC column and also facilitates metabolite ionization in the MS system. In addition to choosing appropriate solvents (such as acetonitrile, methanol, water) and solvent gradients based on the chemical properties of the samples, the addition of eluent additives to suppress unwanted signals or selectively enhance signals of interest can greatly increase the ability to detect and the sensitivity to quantify particular compounds in a mixture. The two most common types of additives are acids and bases (formic acid, ammonium acetate, etc.) to alter pH and compounds such as tributylamine and triethylamine to form ion pairs [38]. The widespread adoption of ultra-performance liquid chromatography (UPLC) in chemical and metabolomic analyses is one prominent development in LC separation technology. Smaller particle size (sub-2 micron) and higher tolerance to back pressure in UPLC systems produce much better resolution in chromatographic separation and greater sensitivity for ion detection than traditional HPLC [19, 39]. In addition, new developments in column chemistry provide more choices such as hydrophilic interaction liquid chromatography (HILIC) columns for separating polar and nonpolar compounds [40]. Nevertheless, reverse phase (RP) columns are still the most commonly used for general LC-MS metabolite analysis.

Ionization of analytes is a prerequisite for mass detection in MS system. Selection of ionization method, such as electrospray ionization (ESI), atmospheric pressure chemical ionization (APCI), and the chemical components of mobile phase have major impacts on ionization efficiency. With regard to mass detectors, two important factors are sensitivity and mass accuracy. Nominal mass MS detectors, such as the triple quadrupole MS, can perform highly selective and sensitive quantitation of targeted metabolites, while detectors with full scan and accurate mass measurement capacities, such as time-of-flight (TOF) or Fourier transform MS [41], have a clear advantage in untargeted metabolomics where simultaneous measurement of many metabolites is required [19].

### Data analysis

For untargeted metabolomic analysis, chromatographic and spectroscopic data generated from LC-MS analysis need to be properly processed before being used in multivariate data analysis (MDA) (Fig. 1). Data processing includes data condensation and reduction by centroiding and deisotoping mass spectra; removal of noise or background signals; and peak identification by setting threshold windows for mass-charge ratio (*m/z*) and retention time (RT) [42]. Furthermore, normalization of MS data against parameters of the whole dataset (such as total ion count, median ion count) or intensities of internal standards (such as creatinine in urine) is commonly conducted to reduce the influence of systematic and sample biases (such as dilution or condensation) [19]. Thus, each unique pair of RT-*m/z* and its signal intensity become the identity of one metabolite. Afterwards, the processed datasets can be either directly used for MDA or further statistically transformed and scaled according to the properties of data and the purpose of MDA.

Both unsupervised MDA, such as principal components analysis (PCA), or supervised MDA, such as projection to latent structures-discriminant analysis (PLS-DA) or orthogonal PLS (OPLS), are widely used to analyze metabolomic data. Compared to traditional statistical methods, such as *t*-test and ANOVA, MDA can better handle and interpret the large datasets generated by LC-MS analysis. After MDA, a large portion of examined dataset is represented by the principle components (PC) in the multivariate models [18]. The sample-PC and sample-sample relationships can be visualized in scores plot of the established MDA model, in which the spatial distance between two samples reflects differences in their chemical composition. In metabolomic analysis of XIT, when a clear separation between samples from vehicle and samples from xenobiotic treatment is observed in the scores plot, the XIT-related metabolites can be conveniently identified in the loadings plot through their correlation with the PCs that separate two treatments (Fig. 1). The chemical identities of biomarkers and metabolites are determined by accurate mass measurement, elemental composition analysis, MS/MS fragmentation and subsequent database searches (such as Human Metabolome Database: http://www.hmdb.ca/, Lipid Maps: http://www.lipidmaps.org/, METLIN database: http://metlin.scripps.edu/). Recent development in bioinformatics has further facilitated metabolite annotation in LC-MS-based metabolomics studies [43, 44].

## Applications of LC-MS based metabolomics of XIT

Metabolic events in XIT encompass biotransformation of xenobiotic (xenobiotic metabolism) and xenobiotic-induced metabolic changes in biological system (Fig. 2). All these metabolic events have distinctive roles in the initiation and progression of XIT. Compared to traditional metabolite analysis, the benefits of adopting LC-MS based metabolomics in studying XIT are mainly based on its analytical capacity to investigate these events effectively. Metabolomic investigation of XIT can be defined as exploratory, such as the identification of new xenobiotic metabolites and biomarkers, or hypothesis-driven, such as the role of enzymes and pathways in XIT (Fig. 2). To achieve these aims, adaptation of effective research approaches is essential in metabolomics studies. In this review, common approaches used in the LC-MS-based metabolomic investigation of XIT-associated xenobiotics metabolism and metabolic changes are examined and their potential applications in resolving practical issues in XIT are illustrated by case studies and proof-of-concept experiments.

### LC-MS-based metabolomic investigation of xenobiotic metabolism

In XIT, biotransformation serves as a double-edged sword. Xenobiotics can be either activated or detoxified by biotransformation reactions catalyzed by xenobiotic metabolizing enzymes (XME) in the body. The balance between bioactivation and detoxification, in many cases, may determine the toxic effects of xenobiotics [1]. Therefore, studying the biotransformation of xenobiotics *in vivo* is essential for understanding and predicting XIT. Since xenobiotic metabolites, especially reactive metabolites, usually are not the most abundant metabolite species in biofluids and tissues, one major challenge in the study of xenobiotic metabolism is how to efficiently and thoroughly identify xenobiotic metabolites among thousands of chemical species in biological samples. Using a radiolabeled xenobiotic to trace its metabolites is a very effective method due to the sensitivity and quantitative nature of radiotracing. However, wide application of radiotracing is hampered by concerns of contamination and health hazards as well as the time and cost associated with the synthesis of radiolabeled compounds.

In recent year, mass defect filtering methods have been developed in the drug metabolism field [45, 46]. Based on high-resolution mass measurement and algorithm-based computation of elemental composition, these metabolite searching methods use the numerical values of mass increase or decrease caused by known metabolism reactions or an artificial mass defect window as the screening filter to identify metabolites formed by single or multiple reactions. The major issue with this approach is that the filters do not cover all *in vivo* biotransformation reactions, especially the many uncommon reactions that may cause dramatic mass changes [47]. In addition, results obtained from mass filtering might be plagued with false-positive entries. Therefore, metabolomics-guided metabolite profiling offers an effective alternative that can circumvent drawbacks and limitations of the abovementioned metabolite identification methods in xenobiotic metabolism research [19].

Samples from diverse sources, including urine, serum, feces, tissue extracts, and *in vitro* incubations, can be used for metabolomic examination of xenobiotic metabolism. In most cases, pooled urine or fecal samples collected within a day are more suitable than blood or tissue samples for profiling xenobiotic metabolites since the majority of xenobiotic compounds do not accumulate in the body. A straightforward approach for identifying *in vivo* xenobiotic metabolites is to conduct metabolomic comparison between samples from vehicle and xenobiotic treatment groups. As xenobiotic and its metabolites only appear in samples from the xenobiotic treatment, it is expected that separation between vehicle and xenobiotic treatment in the metabolomic model should be primarily due to the xenobiotic and its metabolites (Fig. 2). Therefore, analyzing ions that contribute to the separation of xenobiotic and vehicle treatments can lead to the identification of xenobiotic metabolites. Employing this approach, novel metabolites of therapeutic agents, phytochemicals, and dietary compounds, including aminoflavone [48], arecoline [49], fenofibrate [50], melatonin [51], and cocaine [52], have been identified, and novel metabolic routes have been characterized.

Direct metabolomic comparison of vehicle and xenobiotic treatments facilitates the identification of xenobiotic metabolites, especially for the treatments that do not significantly affect endogenous metabolism. However, many xenobiotics also dramatically alter or disrupt endogenous metabolism of carbohydrates, amino acids, and/or lipids. In the metabolomic models of these treatments, metabolites contributing to the separation of vehicle and xenobiotic treatment include both xenobiotic metabolites and the endogenous metabolites responsive to xenobiotic treatment. To avoid interference from endogenous metabolites, a stable isotope-based metabolomic approach using xenobiotics labelled with ^2^H, ^13^C, ^15^N, or ^18^O can be used to facilitate identification of the xenobiotic metabolites. In practice, equal amount of labeled and unlabeled xenobiotic are used in the treatments. Since the endogenous metabolites affected by the labeled or unlabeled xenobiotic treatment are the same or very similar, metabolites contributing to the separation of the labeled and unlabeled groups in the metabolomic models are mainly xenobiotic and its metabolites.

**Figure 3:**
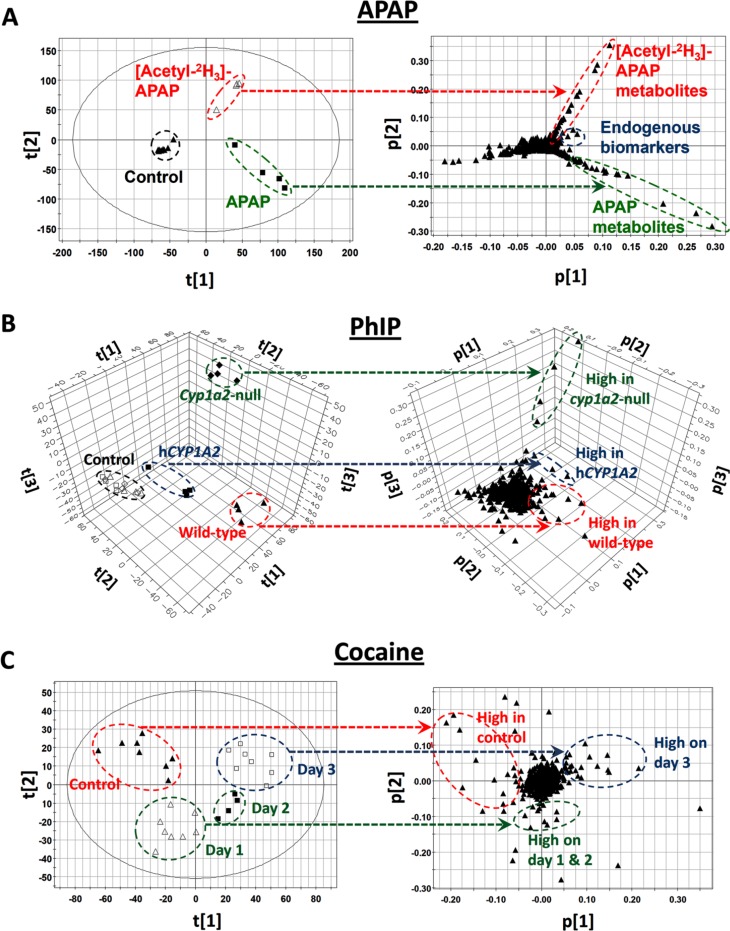
Examples of LC-MS-based metabolomic investigation of metabolic events in XIT. The dashed arrow lines indicate correlations between samples in the scores plot and ions in the loadings plot of multivariate models. ***A**.* Identification of xenobiotic metabolites. Through metabolomic analysis of mouse urine samples from control, APAP, and [Acetyl-^2^H_3_]-APAP treatments, the unlabeled APAP and deuterated APAP metabolites can be conveniently identified in the loadings plot. The ions that increased in both APAP and [Acetyl-^2^H_3_]-APAP treatments are from endogenous metabolism [53]. ***B**.* Role of XME in the biotransformation of xenobiotic. Through the metabolomic analysis of mouse urine samples from PhIP-treated wild-type, *Cyp1a2*-null, and *CYP1A2-*humanized mice, genotype-dependent PhIP metabolism is illustrated by the distinctive distribution of PhIP metabolites in the loadings plot [56]. ***C**.* Kinetics of xenobiotic-induced changes in endogenous metabolism. Through metabolomic analysis of daily mouse serum samples from a 3-day cocaine treatment, lipid species correlating with development of cocaine-induced hepatotoxicity in mice can be identified in the loadings plot [61].

Efficacy of the stable isotope-based approach has been demonstrated in several studies [53, 54]. For example, in a metabolomic investigation of acetaminophen biotransformation in mice, two groups of mice were treated with a hepatotoxic dose of acetaminophen (APAP) and deuterated APAP, respectively. Separation of urine samples from the two treatments in a metabolomic model was mainly caused by APAP metabolites and their labeled counterparts (Fig. 3A). Besides identifying known APAP metabolites in urine, such as cysteinyl-APAP and APAP glucuronide, several new biomarkers that are highly correlated with APAP toxicity were confirmed as APAP metabolites based on the presence of stable isotope in their structures [53]. Another benefit of this approach is to separate the endogenous biomarkers from xenobiotic metabolites in the loadings plot of metabolomic model (Fig. 3A). Furthermore, in a separate study on ethanol-induced hepatotoxicity in mice, a dramatic increase of *N*-acetyl taurine (NAT) in urine was observed after ethanol exposure. However, NAT as a metabolite of ethanol was not confirmed until a metabolomic comparison of urine samples from mice fed unlabeled ethanol and mice fed dueterated ethanol ([^2^H_6_]-ethanol) was performed [54].

In addition to its use in exploratory investigations of novel xenobiotic metabolites, LC-MS-based metabolomics can also be applied to hypothesis-driven investigation of XMEs in XIT when appropriate experiment models are adopted in the studies (Fig. 2). It is known that major XMEs, including functionalizing (phase I) enzymes and conjugating (phase II) enzymes, have different roles in the bioactivation and detoxification of xenobiotics [1]. Animal models containing modified XME genes, such as mutation, knockout, or humanized models, have become powerful tools for investigating the role of XMEs in XIT [55]. Traditional metabolite analyses of xenobiotic metabolism in genetically-modified animal models usually focus on a few metabolites that are considered to be the direct substrates or products of XMEs of interest. However, application of LC-MS-based metabolomics has enabled a much more comprehensive characterization of xenobiotic biotransformations in genetically-modified animal models. For example, the role of CYP1A2 enzymes in the metabolism of 2-amino-1-methyl-6-phenylimidazo[4,5-b]pyridine (PhIP), a widely distributed procarcinogen in the human diet, was defined through a metabolomic comparison of PhIP metabolism in wild-type, *Cyp1a2*-null, and *CYP1A2*-humanized mice (Fig. 3B). Comprehensive profiling of PhIP metabolites in these three mouse lines not only confirmed the catalytic activity of CYP1A2 in PhIP metabolism, but also revealed human-mouse interspecies differences as well as the involvement of other P450s in PhIP metabolism [56].

### LC-MS-based metabolomic investigation of xenobiotic-induced metabolic changes in biological systems

Disruption of metabolic systems always occurs in XIT. Metabolic changes can be immediate and direct effects of chemical exposure, or delayed and side effects of XIT. Characterizing these changes in metabolic systems is essential for understanding the molecular mechanisms of XIT since the disorders in energy and nutrient metabolism are usually the most prominent phenotypes of XIT. Targeted metabolite analyses in XIT studies are usually highly subjective due to their typical focus on suspected metabolites, or guided by observed changes in gene and protein levels. In contrast, untargeted metabolomic analysis can simultaneously monitor diverse metabolic changes in biological systems, and provide guidance for mechanistic investigations at the enzyme, protein, and gene levels [57].

Choosing an appropriate sample source for metabolomic analysis is important in order to obtain meaningful information on XIT-associated metabolic changes because each source has a different mixture of metabolites which represents the different metabolic activities associated with the source. Possible sample sources include biofluids, excreta, tissues, cell pellets, and cell culture media. In animal experiments, urine and fecal samples reflect the consummate effects of XIT-induced metabolic activities, while biofluids and tissue samples reflect the real-time metabolic status in XIT. In addition, metabolomic analysis of tissue/organ targets of xenobiotics can identify local or tissue specific effects of xenobiotics on metabolism while urine and serum can reflect the systemic impact of tissue toxicity on the whole body.

Similar to the metabolomic investigation of xenobiotic metabolism, a straightforward approach in the metabolomic investigation of XIT-induced metabolic changes is to determine differences between untreated and treated subjects or differences between susceptible and resistant subjects. Based on the pathophysiological and metabolic phenotypes of XIT, such as fatty liver, muscle degradation, and oxidative stress, metabolomic analysis can be adjusted to focus on specific classes of metabolites, including lipids, amino acids, and organic acids. For example, changes in the composition and concentrations of lipid species are observed in many XIT events. However, the significance of these changes in lipidome is not well understood. GC-based fatty acid profiling was the most widely performed lipid analysis, but this assay is insufficient to reflect the real biochemical consequences caused by the disruption of lipid metabolism since bioactive lipid species are usually complex lipids [58]. Compared to GC-based fatty acid analysis, LC-MS-based lipidomics has clear advantages in detecting complex lipid species, which makes it an ideal tool for the simultaneous examination of diverse lipid species, including XIT-induced changes in lipid metabolism [59, 60]. In a recent LC-MS-based lipidomics study of cocaine-induced liver injury, progression of hepatotoxicity in a 3-day cocaine treatment was closely associated with disruption of serum lipidome since the time-dependent separation of serum samples in a MDA model represented the contribution of different lipid species on each day of cocaine exposure (Fig. 3C) [61]. Guided by this lipidomic model and MS-based structural elucidation, accumulation of long-chain acylcarnitines was defined as a prominent cocaine-induced metabolic change. Because of the importance of long-chain acylcarnitines in mitochondrial fatty acid catabolism, this observation led to the identification of cocaine-induced inhibition of fatty acid oxidation in the liver. The relevance of this observation to cocaine-induced hepatotoxicity was further validated by cotreatment with fenofibrate, which activated peroxisome proliferator-activated receptor α (PPARα), a central regulator of fatty acid oxidation, and protected the mice against toxicity. Furthermore, LC-MS-based lipidomics revealed that cotreatment with the PPARα ligand reversed cocaine-induced changes in the lipidome [61]. Besides lipids, chemical-induced changes in amino acid metabolism, antioxidant turnover, and carbohydrate metabolism, have also been effectively examined by LC-MS-based metabolomics [62–64].

As a significant component of biological system, gut flora preform metabolic reactions that differ from their host, producing both nutrients and non-nutrients [65]. A broad MS-based metabolomics study that used GC-MS and LC-MS analyses, of intestinal digesta from conventional and germ-free mice revealed the significant contribution of bacterial metabolites to mammalian blood metabolites [66]. In addition to their known effects in intestinal ailments, metabolic diseases, and immune diseases [67–69], gut flora can also affect XIT through indirect regulation of XMEs [70] or direct interference of xenobiotic metabolism [71, 72]. Because of the complexity and unpredictability of bacterial metabolism, it is expected that LC-MS-based metabolomics should be more effective than traditional metabolite analysis for examining the influences of xenobiotics on symbiotic gut flora in humans and animals. For example, LC-MS-based metabolomics of ethanol treatment has shown that the development of ethanol-induced fatty liver was associated with increased bacterial metabolites in urine [54]. Expanding the application of metabolomics in studies of microflora metabolism will generate more insights into the roles of gut flora in XIT.

Besides its usage in exploratory investigations to identify biomarkers and new metabolites, LC-MS-based metabolomics can also be applied to hypothesis-driven investigations of metabolic pathways in XIT-induced metabolic changes (Fig. 2). Hypotheses of the roles of specific metabolizing enzymes and regulatory pathways in XIT can be tested through a combination of metabolomic analysis with other experimental approaches. For example, animal models that have different sensitivities to xenobiotic exposure or that are genetically altered to interfere with XIT can be compared. For example, dextran sulfate sodium (DSS)-induced acute colitis was examined by LC-MS-based serum metabolomic analysis. Inhibition of stearoyl-CoA desaturase 1 (SCD1), an enzyme responsible for converting saturated fatty acids to mono-unsaturated fatty acids, was identified after observing an increased ratio of stearoyl lysophosphatidylcholine to oleoyl lysophosphatidylcholine in the serum of DSS-treated mice. The anti-inflammatory role of SCD1 in DSS-induced colitis was further defined by LC-MS-based metabolomics and biochemical analyses of the relationship between SCD1 activity and DSS-induced proinflammatory effects [73].

Examining the roles of metabolizing enzymes and regulatory pathways in XIT is just one application of LC-MS-based metabolomics in the hypothesis-driven investigation of XIT-related metabolic changes. Determining the metabolic routes contributing to observed metabolic phenotypes or specific changes in small-molecule biomarkers in XIT, such as the upstream and downstream metabolites of identified biomarkers, is another potential application of hypothesis-driven metabolomic investigation (Fig. 2). When a hypothesis about the source and metabolic route of a biomarker is proposed, a combination of LC-MS-based metabolomics and stable isotope tracer can become a powerful analytical tool to test the hypothesis. The techniques of using stable isotope-labeled glucose, amino acids, and fatty acids to interrogate the metabolic networks have been widely used in studying xenobiotic-induced metabolic changes [74]. The methodology of LC-MS-based untargeted metabolomics with stable isotope tracer has been improved recently [75]. Therefore, it is reasonable to believe that application of LC-MS-based metabolomics and stable isotope tracer will generate more mechanistic insights into XIT-related metabolic changes in future.

## Conclusion

Wide adoption of metabolomics in biomedical research in recent years has demonstrated its advantages over traditional metabolite analysis approaches. As a branch of metabolomic techniques, LC-MS-based metabolomics possesses great promise for becoming the most commonly used analytical platform to identify novel metabolites and elucidate metabolic changes due to its versatility and sensitivity. As illustrated by the case studies in this review, LC-MS-based metabolomics has merits in unraveling novel information on the metabolic alterations caused by XIT and the underlying mechanisms responsible for these alterations. With the development of new LC-MS techniques and data analysis methods, LC-MS-based metabolomics will have more applications in both exploratory and hypothesis-driven investigations of XIT.

## Abbreviations

APAP, Acetaminophen; GC-MS, gas chromatography-mass spectrometry; HILIC, hydrophilic interaction liquid chromatography; HPLC, high-performance liquid chromatography; LC-MS, liquid chromatography-mass spectrometry; MDA, multivariate data analysis; MS, mass spectrometry; MDA, multivariate data analysis; NMR, nuclear magnetic resonance; OPLS, orthogonal PLS; PCA, principal components analysis; PhIP, 2-amino-1-methyl-6-phenylimidazo[4,5-b]pyridine; PLS-DA, projection to latent structures-discriminant analysis; PPAR., peroxisome proliferator-activated receptor alpha; UPLC, ultra-performance liquid chromatography; XIT, xenobiotic-induced toxicity; XME, xenobiotic metabolizing enzyme.
